# Gut Microbial Tryptophan Metabolism Is Involved in Post‐Cardiac Arrest Brain Injury via Pyroptosis Modulation

**DOI:** 10.1111/cns.70381

**Published:** 2025-04-22

**Authors:** Chenghao Wu, Mengyuan Diao, Shuhang Yu, Shaosong Xi, Zhipeng Zheng, Yang Cao, Shuai Wang, Ying Zhu, Mao Zhang, Wei Hu

**Affiliations:** ^1^ Department of Critical Care Medicine, Affiliated Hangzhou First People's Hospital, School of Medicine Westlake University Hangzhou China; ^2^ Department of Emergency Medicine, Second Affiliated Hospital Zhejiang University School of Medicine Hangzhou China; ^3^ Anesthesia Center of Critical Care Research, Department of Anesthesia, Critical Care and Pain Medicine, Massachusetts General Hospital Harvard Medical School Boston MA USA; ^4^ Department of Intensive Care Unit, Second Affiliated Hospital Zhejiang University School of Medicine Hangzhou China; ^5^ Department of Pulmonary and Critical Care Medicine, Sir Run Run Shaw Hospital Zhejiang University School of Medicine Hangzhou China; ^6^ Department of Neurosurgery, Affiliated Hangzhou First People's Hospital, School of Medicine Westlake University Hangzhou China

**Keywords:** aryl hydrocarbon receptor, cardiac arrest, cerebral ischemia–reperfusion injury, gut microbiota, L‐kynurenine, pyroptosis

## Abstract

**Aims:**

Post‐cardiac arrest brain injury (PCABI) is a leading cause of death in cardiac arrest/cardiopulmonary resuscitation (CA/CPR) victims and long‐term disability in CA/CPR survivors. Despite previous evidence indicating that the microbiota‐gut‐brain axis is critically involved in many neurological disorders, no research has hitherto established a connection between the gut microbiota and PCABI through this axis. This study aims to explore the biological roles of microbial tryptophan metabolites in the progression of PCABI.

**Methods:**

To achieve this, we pretreated rats with a cocktail of broad‐spectrum antibiotics (Abx) to eradicate the gut microbiota before establishing a 7‐min asphyxia‐CA/CPR model.

**Results:**

Remarkably, the 24‐h survival rate and neurological outcomes improved in Abx/CPR rats. Fecal 16s rDNA sequencing and PICRUSt2 analysis revealed that Abx reshaped the microbial community and elevated the proportion of microbial tryptophan metabolism in rats. Metabolomic profiling suggested that Abx shifted the phenotype of microbial tryptophan metabolism from the indole pathway to the kynurenine pathway, thereby increasing the levels of the neuroprotective metabolite kynurenine in the feces, circulation, and ultimately the brain. Furthermore, the hippocampal expression of aryl hydrocarbon receptor (AhR), an endogenous receptor of kynurenine, was upregulated in Abx/CPR rats. In vitro experiments further demonstrated that the neuroprotective effects of kynurenine are AhR‐dependent and that AhR activation could negatively regulate the NLRP3 protein expression. Supporting this, results from qRT–PCR, immunohistochemistry, and immunofluorescence in the rat cerebral cortex exhibited that L‐kynurenine inhibited NLRP3‐induced pyroptosis.

**Conclusions:**

Our study provides a direct clue to the essential participation of the microbiota‐gut‐brain axis in the progression of PCABI. It demonstrates that kynurenine might attenuate PCABI by inhibiting NLRP3‐induced pyroptosis.

## Graphical abstract

(A) The increase of gut microbiota‐mediated kynurenine serves as a component of a range of self‐protective responses post‐resuscitation. (B) Antibiotics pretreatment further increases the levels of gut microbiota‐mediated kynurenine, which attenuates PCABI to a greater extent. (C) Systematic supplementation of L‐Kyn might be a potential therapeutic strategy for PCABI.

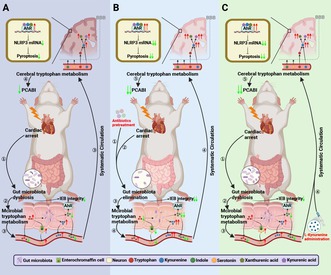

## 1 Introduction

Cardiac arrest (CA) is a global issue and frequently has a sudden onset with high mortality [1, 2]. Even in cases where initial cardiopulmonary resuscitation (CPR) is successful, up to 70% of survivors die of post‐cardiac arrest brain injury (PCABI) [3]. PCABI consists of ischemic and reperfusion injury, which occurs sequentially during CA (no perfusion), CPR (hypoperfusion), and post‐resuscitation (early hyperperfusion and delayed hypoperfusion) phases [4]. To date, no direct treatment has been found for PCABI [5]. Therefore, further study of the pathogenetic mechanism underlying PCABI and its effective therapeutic targets is urgent.

Accumulating data points to a critical role of gut microbiota in the bidirectional communication between the gastrointestinal tract and brain, namely, the microbiota‐gut‐brain axis [6, 7]. For investigating microbe‐host relationships, a cocktail of broad‐spectrum non‐absorbable antibiotics (Abx) is one of the most widely used tools, which offers better temporal flexibility and has better translatability to human situations [8, 9]. Numerous effects of the gut microbiota on the microbiota‐gut‐brain axis are mediated by microbial metabolites [10]. Notably, as a key node integrating gut microbiota and brain function, tryptophan (Trp) metabolism has been described in various neurological diseases, including Huntington's disease, Alzheimer's disease, migraine, and stroke [11]. However, whether and how microbial Trp metabolism is involved in the progression of PCABI remains unknown.

Trp metabolism follows three major pathways: kynurenine (Kyn) pathway (KP), indole pathway (IP), and serotonin pathway (SP) (Figure S1) [12]. The IP is mainly regulated by gut microbiota, and the SP predominantly occurs in gut‐resident enterochromaffin cells [13], while the KP is the major route of Trp metabolism in the mammalian brain and degrades 95% of the free Trp [7]. The brain contains a set of KP enzymes, including indoleamine 2,3‐dioxygenase (IDO), kynurenine 3‐monooxygenase (KMO), and kynurenine aminotransferase 2 (KAT2) [14]. The KP metabolites exhibit distinct neuroactive properties, including Kyn (neuroprotectant), kynurenic acid (KynA; neuroprotectant), xanthurenic acid (XA; antioxidant), and quinolinic acid (QA; neurotoxin) [14]. As Kyn and Trp readily penetrate the blood–brain barrier (BBB), fluctuations in the peripheral levels of Trp and Kyn could directly affect cerebral KP [15]. Indeed, approximately 60% of cerebral KP metabolism is initiated by the peripheral Kyn [16]. However, KynA, QA, and XA show poor BBB penetration and must be synthesized within the brain [15]. A recent study indicated that elevated levels of peripheral Kyn might predict unfavorable outcomes in patients with PCABI [17], although the underlying mechanism remains unclear.

Previous reports have suggested that the neuroprotective effects of Kyn, KynA, and XA are attributed to their role as endogenous ligands of the aryl hydrocarbon receptor (AhR) [18, 19, 20] which modulates the immune response, inflammatory reactions, and tissue repair [21, 22, 23]. Several recent studies have reported that the AhR inhibits inflammasome activation [24, 25]. The inflammasomes are assembled by NOD‐like receptor family protein 3 (NLRP3), apoptosis‐associated speck‐like proteins containing a CARD (ASC), and caspase‐1, which is crucial for the induction of pyroptosis and the release of pro‐inflammatory cytokines [26]. Emerging evidence suggests that pyroptosis is involved in the pathogenesis of PCABI, and the inhibition of pyroptosis improves neurological dysfunction post‐resuscitation [27, 28]. Furthermore, many AhR modulators are under development [11], including CH‐223191 (CH; AhR antagonist) and L‐kynurenine (L‐Kyn; putative AhR agonist). Thus, the inhibition of pyroptosis with AhR agonists may serve as a potential therapeutic strategy for PCABI.

Based on the results of the aforementioned studies, we aim to reveal the comprehensive role of microbial Trp metabolism in PCABI. We found that 4‐week Abx pretreatment dramatically improved the survival and neurological outcomes of Abx/CPR rats. Integrative analysis of multi‐omics and in vitro mechanistic experiments indicated that gut microbial Trp metabolites‐mediated AhR activation could partially attenuate PCABI by inhibiting inflammasome‐induced pyroptosis, which offers novel potential therapeutic targets.

## 2 Materials and Methods

Detailed methods are available in the Supporting Information.

### 2.1 Statistical Analysis

GraphPad Prism 9.0 Software (GraphPad, USA) was employed to conduct statistical analyses and plot graphs. Normality was assessed by the Kolmogorov–Smirnov test, and homogeneity of variances was tested via the Brown–Forsythe test. For normally distributed datasets with equal variances, unpaired Student's *t*‐test and one‐way analysis of variance (ANOVA) were used to determine the difference between two groups and between multiple groups, respectively. For normality‐distributed datasets with unequal variances, Welch's *t*‐test and Welch's ANOVA were used to determine the difference between two groups and between multiple groups, respectively. For non‐normally distributed datasets, nonparametric alternatives were applied as follows: Mann–Whitney *U* test (two groups) or Kruskal–Wallis test (multiple groups). Correlations between continuous variables were evaluated by Pearson's test (normal) or Spearman's rank correlation (non‐normal), and categorical variables were compared using Fisher's exact test. The cumulative survival rate was analyzed by a Kaplan–Meier curve, and *p* values were assessed with a log‐rank (Mantel–Cox) test. Continuous variables are expressed as the mean ± standard deviation (normal) or median with interquartile range (non‐normal). *p* Values < 0.05 were considered statistically significant.

## 3 Results

### 3.1 Gut Microbiota Was Associated With Survival and Neurological Outcomes in CA/CPR Rat

**FIGURE 1:**
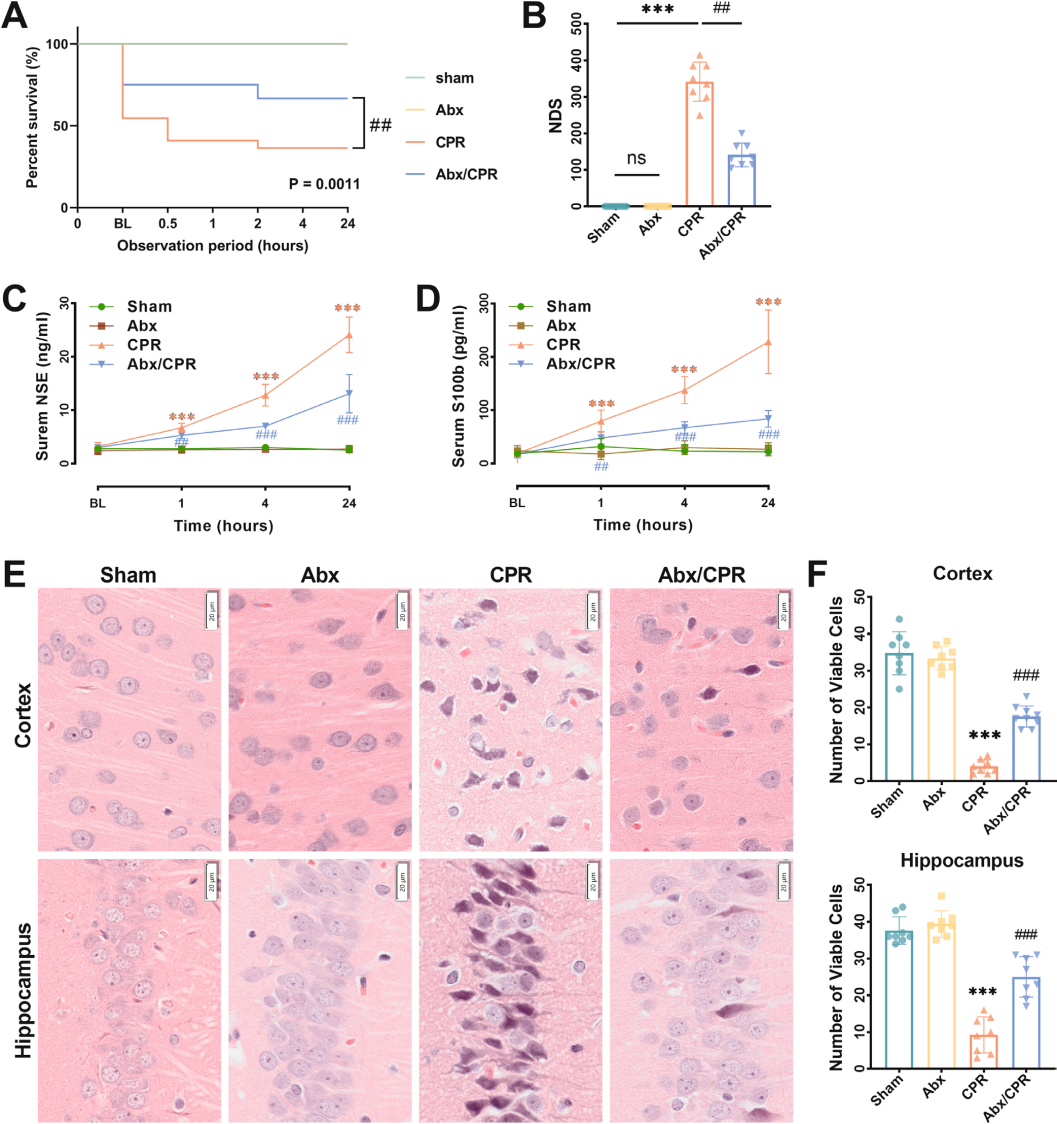
Survival and neurological outcomes were improved in Abx/CPR rats. (A) Cumulative survival analysis (a Kaplan–Meier curve). (B) NDS of survived rats at 24 h post‐resuscitation (*n* = 8). (C, D) Serum concentrations of NSE and S100b at BL and 1, 4, and 24 h post‐resuscitation (*n* = 8). (E, F) Representative images of hematoxylin–eosin stain (scale bar = 20 μm), and quantification of viable neurons (*n* = 8), in the hippocampus CA1 region and precentral gyrus of the frontal lobe at 24 h post‐resuscitation. Data are expressed as mean ± SD. ***p < 0.001 versus sham group; ^##^ *p* < 0.01, ^###^ *p* < 0.001 versus CPR group; ns, not significant.

Fifty rats were allocated to Abx pretreatment (*n* = 20) or control (*n* = 30). Among these, 12/20 Abx‐pretreated rats and 22/30 control rats successfully underwent CA/CPR (Figure S2A), with survival rates calculated from these initial cohorts. All CA/CPR animals were monitored for 24 h, and Kaplan–Meier analysis revealed a significantly higher cumulative survival rate in Abx/CPR rats compared to controls (log‐rank test, *p* = 0.0011; Figure 1A). Moreover, the time from asphyxia to CA was prolonged and the time required for the restoration of spontaneous circulation (ROSC) was shortened in Abx/CPR rats versus CPR rats (Table S3). For the heart rate (HR), at 0.5 h post‐resuscitation, the HR decreased in CPR rats versus sham rats, while this change was restored in Abx/CPR rats (Table S3). For mean arterial pressure (MAP), at 0.5‐, 1‐, 2‐, and 4‐h post‐resuscitation, the MAP in CPR rats was lower than in sham rats. At 1‐ and 4‐h post‐resuscitation, MAP in Abx/CPR rats was higher than in CPR rats (Table S3). For End‐tidal CO_2_ (EtCO_2_), at 0.5‐, 1‐, 2‐, and 4‐h post‐resuscitation, EtCO_2_ increased sharply in CPR rats versus the sham rats, while the EtCO_2_ of the Abx/CPR rats was consistently below baseline (Table S3).

To estimate the neurological deficits, the Neurologic deficit score (NDS) was measured at 24 h post‐resuscitation. The results showed a notable rise in NDS in both the CPR and Abx/CPR rats compared to sham rats. However, the NDS decreased dramatically in the Abx/CPR rats versus the CPR rats (Figure 1B). Furthermore, at 1, 4, and 24 h post‐resuscitation, the serum neuron‐specific enolase (NSE) and S100 protein b (S100b) contents were incrementally elevated in CPR and Abx/CPR rats, whereas these increments were lessened in the Abx/CPR rats versus the CPR rats (Figure 1C,D). H&E staining revealed that Abx significantly augmented viable neuron counts in the cornu ammonis 1 (CA1) region of the hippocampus and the precentral gyrus of the frontal lobe (Figure 1E,F). Overall, these data demonstrate that Abx pretreatment attenuates PCABI and improves neurological outcomes in rats.

### 3.2 Microbial Trp Metabolism Is Involved in the Pathophysiology of PCABI

**FIGURE 2:**
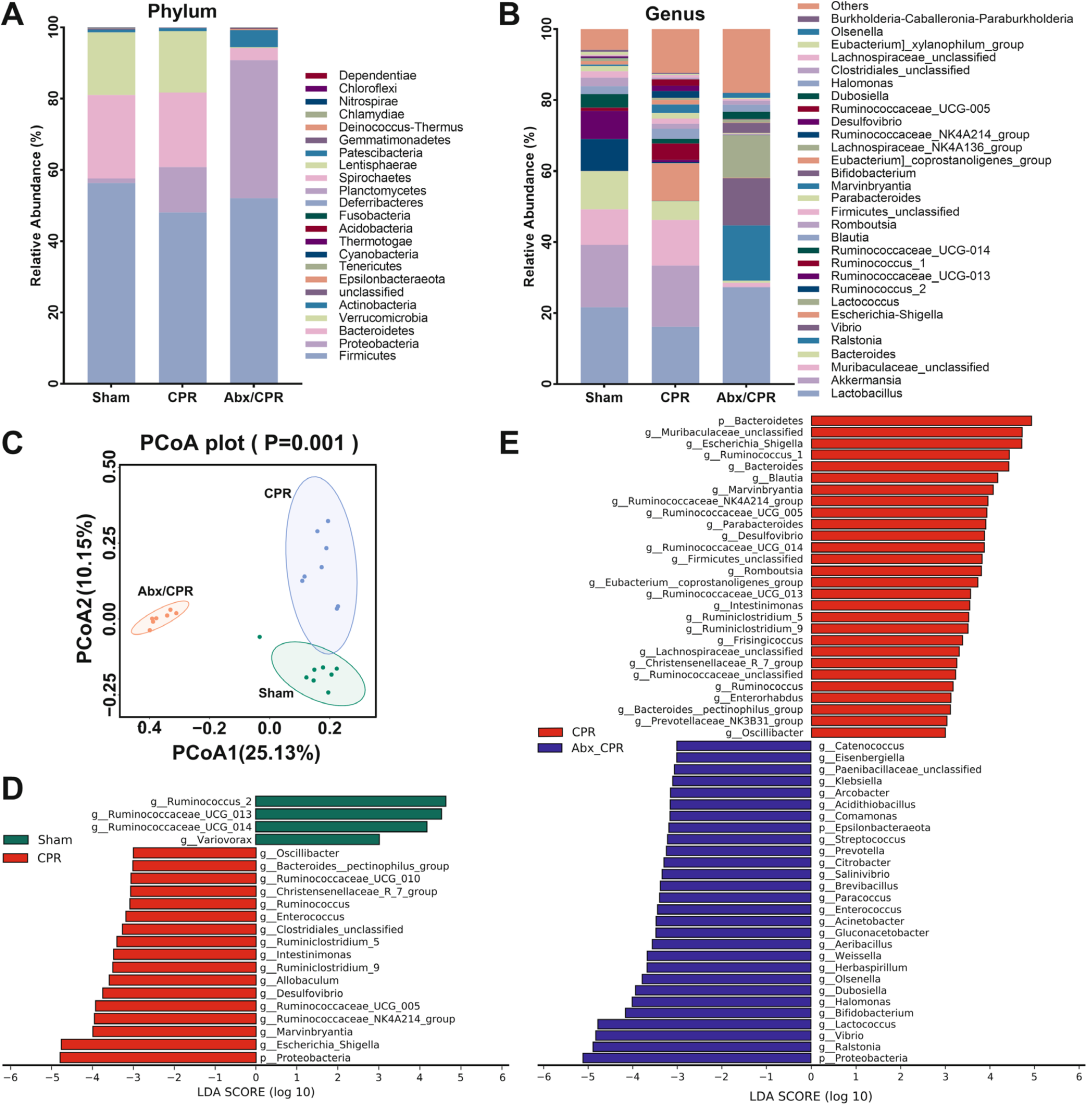
Microbial composition was altered in Abx/CPR rats. (A, B) Taxonomic profiling at the phylum and genus levels (*n* = 7–8). (C) Microbial β‐diversity (PCoA with unweighted UniFrac distance; *n* = 7–8). (D, E) Differential abundance microbiota (LDA score > 3.0) obtained from LEfSe analysis (*n* = 7–8).

As the effects of Abx on CA/CPR implied the crucial roles of gut microbiota in PCABI progression, we next characterized the microbial composition and function to elucidate the underlying mechanisms. The results showed that four‐week Abx pretreatment sharply decreased the total bacterial load (Figure S3A). Thereafter, 16S rDNA sequencing was conducted to identify the microbial composition. At the phylum level, the gut microbiota across groups were dominated by *Firmicutes*, *Proteobacteria*, *Bacteroidetes*, and *Verrucomicrobia* (Figure 2A). At the genus level, the gut microbiota across groups were dominated by *Lactobacillus*, *Akkermansia*, *Muribaculaceae_unclassified*, *Bacteroides*, *Ralstonia*, *Vibrio*, *Escherichia‐Shigella*, *Lactococcus*, *Ruminococcus_2*, and *Ruminococcus_UCG‐013* (Figure 2B). The Chao1 index (α‐diversity: diversity within habitats) dramatically decreased in Abx/CPR rats versus CPR rats, with no statistical differences between CPR and sham rats (Figure S3B). Principal coordinates analysis (PCoA) with unweighted UniFrac distance (β‐diversity: diversity among habitats) revealed distinct microbial composition among the three groups (Figure 2C). Subsequently, a linear discriminant analysis (LDA) effect size (LEfSe) analysis was conducted to distinguish the differentially enriched microbes between the groups (Figure 2D,E).

**FIGURE 3:**
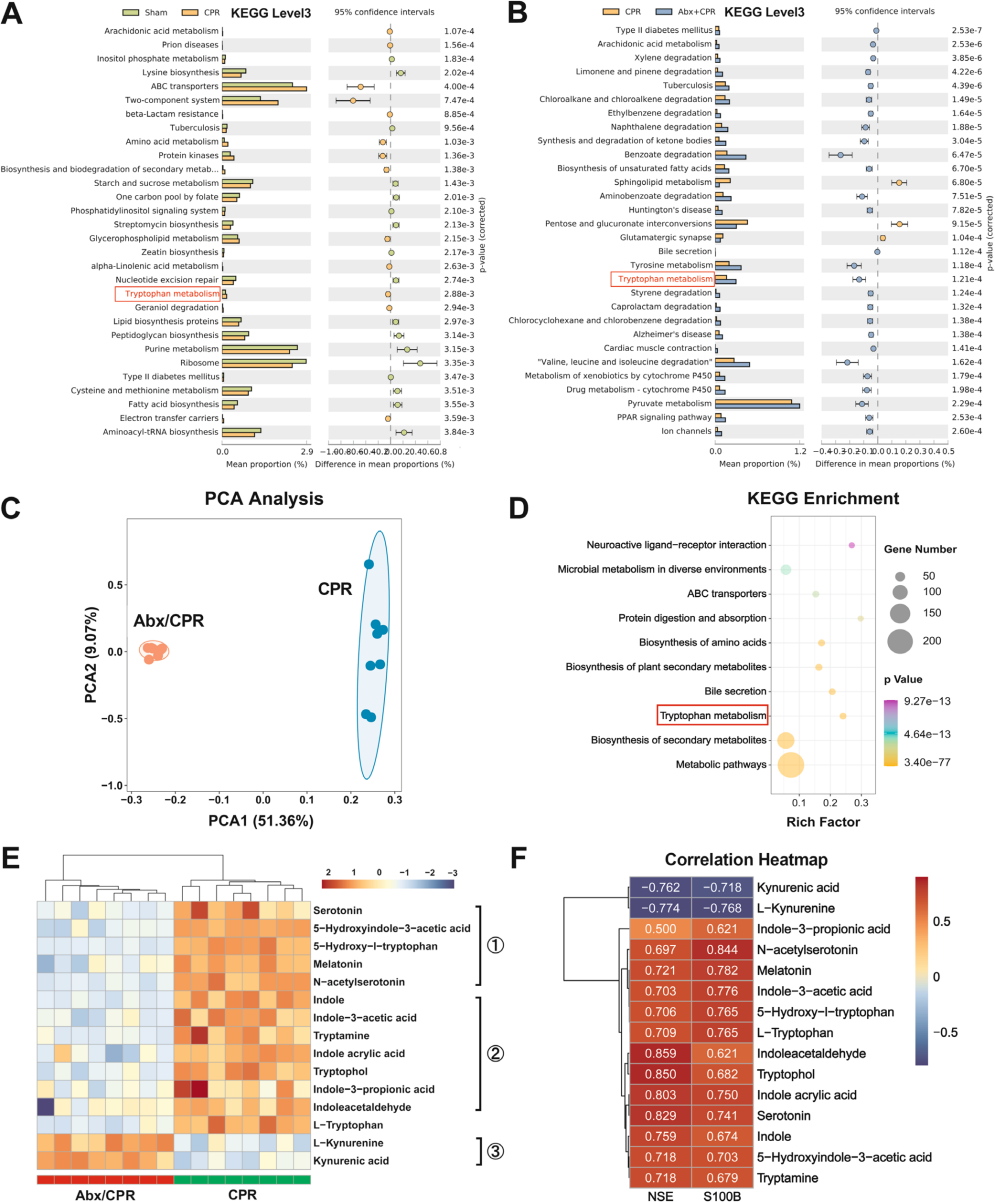
The phenotype of gut microbial Trp metabolism was shifted in rats. (A, B) Predicted functional content at KEGG level 3 (top 30) between two groups. Data are presented as mean proportion and its 95% confidence intervals only with the difference of mean proportions > 0.12%, a ratio of proportion > 2, and *p* < 0.01 between the two groups (*n* = 7–8). (C) PCA of the fecal metabolites (*n* = 8). (D) Statistics of KEGG enrichment of fecal metabolites (*n* = 8). (E) Heatmap of microbial tryptophan metabolites (① serotonin pathway, ② indole pathway, ③ Kyn pathway; *n* = 8). (F) Correlation analysis between differential metabolites and biochemical index (Spearman's *ρ* > 0.5 or < −0.5 and *p* < 0.05; *n* = 8).

As compositional alterations affect gut microbial functions, we next assessed the functional profiles of gut microbiota by Phylogenetic Investigation of Communities by Reconstruction of Unobserved States 2 (PICRUSt2) analysis. Compared to sham rats, the mean proportion of the metabolism pathway decreased at Kyoto Encyclopedia of Genes and Genomes (KEGG) level 1, while that of the neurodegenerative disease pathway increased at KEGG level 2 in CPR rats (Figure S4A). In contrast, compared to CPR rats, the mean proportion of the metabolism pathway was not significantly different at KEGG level 1, but the neurodegenerative disease pathway decreased at KEGG level 2 in Abx/CPR rats (Figure S4B). Remarkably, at KEGG level 3, the microbial Trp metabolism increased proportionally in CPR rats versus sham rats and further escalated in Abx/CPR rats versus CPR rats, indicating its involvement in PCABI pathophysiology and potentially relating to the protective effect of Abx pretreatment on PCABI (Figure 3A,B).

### 3.3 Abx Pretreatment Shifted the Phenotype of Gut Microbial Trp Metabolism in Rats

PICRUSt2 analysis showed significant enrichment of Trp metabolism in gut microbiota; considering this, we next conducted an untargeted metabolomic analysis to detect fecal metabolic profiles. PCA revealed a clear separation of microbial metabolites between Abx/CPR rats and CPR rats (Figure 3C). KEGG pathway enrichment analysis showed intensive associations of the differential metabolites with neuroactive ligand‐receptor interaction, biosynthesis of amino acids, and Trp metabolism, aligning with the PICRUSt2 analysis (Figure 3D).

Trp metabolites emerged as key differential substances in the feces of Abx/CPR and CPR rats (Figure 3E). Specifically, the fecal SP metabolites, including serotonin, N‐acetyl‐serotonin, 5‐hydroxyindole‐3‐acetic acid, 5‐hydroxy‐l‐tryptophan, and melatonin, were significantly decreased in Abx/CPR rats versus CPR rats, likely due to reduced host excretion since more than 90% of the body's serotonin is produced in intestinal enterochromaffin cells [12]. The IP metabolites like indole, indole‐3‐acetic acid (IAA), tryptamine, indole acrylic acid (IA), tryptophol, indole‐3‐propionic acid (IPA), and indole acetaldehyde (IAAld) were also downregulated in Abx/CPR rats versus CPR rats. Conversely, the KP metabolites Kyn and KynA were upregulated in Abx/CPR rats versus CPR rats. Furthermore, Abx pretreatment did not affect the body weight of rats (Table S3), suggesting the practically equivalent dietary Trp intake. Thus, the downregulation of fecal Trp in Abx/CPR rats is attributed to increased host absorption.

Moreover, the association analysis of fecal Trp metabolites and serum biochemical markers showed that NSE and S100b negatively correlated with the KP metabolites (Kyn and KynA) but positively correlated with the IP metabolites (indole, IAA, tryptamine, IA, tryptophol, IPA, IAAld) (Figure 3F). These results demonstrate that Abx shifts the phenotype of gut microbial Trp metabolism from IP to KP and may impact neurological outcomes via modulation of Trp metabolites.

### 3.4 Alterations in Microbial KP Metabolites Might Affect Cerebral KP

**FIGURE 4:**
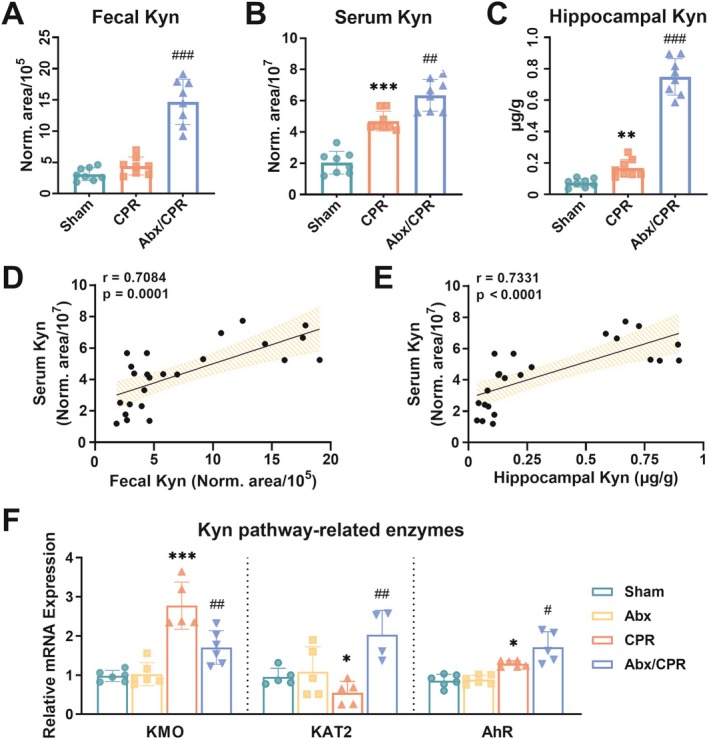
Alterations in microbial KP metabolites might affect cerebral KP. (A–C) Alterations in fecal Kyn, serum Kyn, and hippocampal Kyn (*n* = 8). (D) Pearson's correlation analysis between fecal Kyn and serum Kyn (*n* = 24). (E) Pearson's correlation analysis between hippocampal Kyn and serum Kyn (*n* = 24). (F) Hippocampal gene expression of KAT2, KMO, and AhR (*n* = 4–6). Data are expressed as mean ± SD. *p < 0.05, **p < 0.01, and ***p < 0.001 versus sham group; ^#^ *p* < 0.05, ^##^ *p* < 0.01, and ^###^ *p* < 0.001 versus CPR group.

As Trp and Kyn can readily penetrate the BBB, we next detected serum Trp and Kyn levels and conducted hippocampal Trp metabolic profiling (Figure S5A). Specifically, CA/CPR resulted in significantly elevated levels of fecal Trp and a downward trend in serum Trp compared to sham rats; Abx restored these changes (Figure S5B,C). Additionally, CA/CPR induced an upward trend of fecal Kyn and a sharp rise in serum Kyn compared to sham rats, and Abx further raised serum and fecal Kyn levels (Figure 4A,B). Serum Trp negatively correlated with fecal Trp (Figure S5E), while serum Kyn positively correlated with fecal Kyn (Figure 4D). Regarding hippocampal Trp metabolic profiling, the levels of Kyn, KynA, and QA increased, whereas the levels of Trp, nicotinamide adenine dinucleotide (NAD), and serotonin decreased in CPR versus sham rats. Levels of Trp, Kyn, KynA, XA, NAD, and 5‐hydroxyindole‐3‐acetic acid increased, while the levels of serotonin and QA decreased in Abx/CPR versus CPR rats (Figure 4C and Figure S5A,D). Trp and Kyn in serum were positively associated with Trp and Kyn in the hippocampus, respectively (Figure 4E and Figure S5F). The hippocampal and peripheral IDO activity in CPR rats was significantly higher than in sham rats (Figure S5G). Further, CA/CPR induced decreased KMO gene expression and increased KAT2 gene expression compared to sham rats, whereas Abx/CPR reversed these trends (Figure 4F). Overall, CA/CPR and Abx induced a series of alterations in the KP metabolites and enzymes.

### 3.5 The Neuroprotective Effects of L‐Kyn Might Be AhR‐Dependent

**FIGURE 5:**
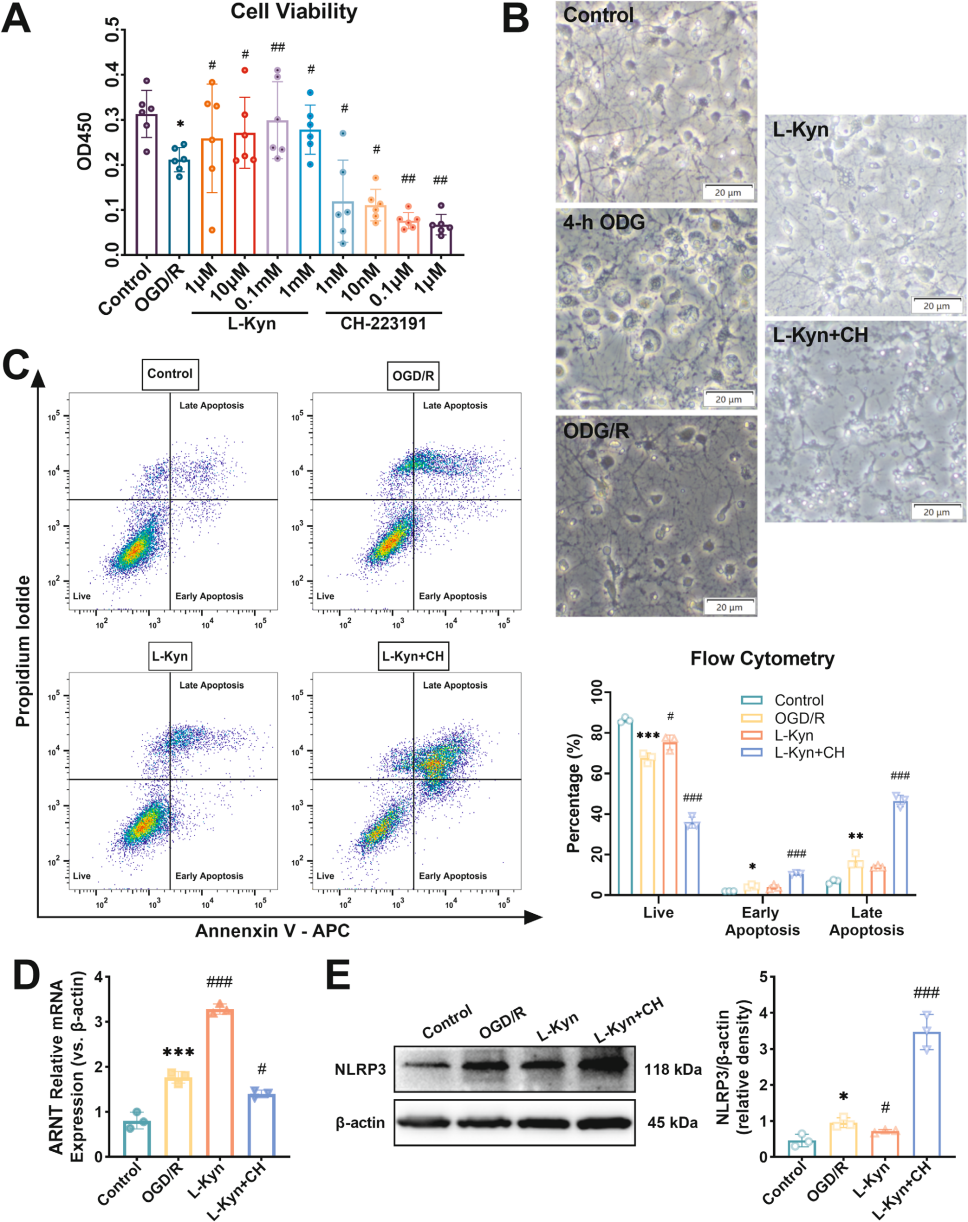
AhR activation negatively regulated NLRP3 protein expression in the OGD/R neurons. (A) Neuronal cell viability (*n* = 6). (B) Morphological changes of neurons (scale bars = 20 μm). (C) Neuronal apoptosis was measured by flow cytometry of Annexin V/PI staining (*n* = 3). Quantitative data were shown as in the bar graph. (D) Relative mRNA expression of ARNT (*n* = 3). (E) Relative NLRP3 protein levels of neurons. Quantitative data were shown as in the bar graph. Data are representative of three independent experiments (*n* = 3 biological replicates). Blot images have been cropped to improve clarity; the raw images of blots are shown in Supporting Information. Data are presented as mean ± SD. *p < 0.05, **p < 0.01, and ***p < 0.001 versus control group; ^#^ *p* < 0.05, ^##^ *p* < 0.01, and ^###^ *p* < 0.001 versus OGD/R group.

The neuroprotective property of Kyn is mostly facilitated through its capacity as an agonist for AhR. We found that the hippocampal AhR mRNA expression was elevated in CPR rats and further upregulated in Abx/CPR rats compared to sham rats (Figure 4F). For AhR‐targeted regulation, the primary neuronal cultures were subjected to an oxygen–glucose deprivation/reoxygenation (OGD/R) model and treated with the concentration gradient of L‐Kyn or CH, identifying the relatively suitable concentration of L‐Kyn (0.1 mM) and CH (1 μM) (Figure 5A). For cell morphology analysis, the neurons in each treatment group showed varying degrees of pyroptosis features (Figure 5B). Specifically, neurons in the 4‐h OGD group showed cellular swelling with bubbles [26]. After 24‐h reoxygenation, neurons exhibited membrane rupture and leakage of cell content [26].

Flow cytometry demonstrated that the percentage of live cells increased in L‐Kyn‐treated neurons but decreased in L‐Kyn + CH‐treated neurons compared to OGD/R neurons. The percentages of early and late apoptotic cells were decreased in L‐Kyn‐treated neurons but increased in L‐Kyn + CH‐treated neurons compared to OGD/R neurons (Figure 5C). Moreover, OGD/R exposure and L‐Kyn treatment increased the gene expression of aryl hydrocarbon receptor nuclear translocator (ARNT), while CH decreased the gene expression level of ARNT induced by L‐Kyn (Figure 5D). An elevated expression of ARNT is correlated with the activation and nuclear translocation of AhR [23]. Notably, OGD/R exposure induced a significant rise in NLRP3 protein expression, which was downregulated by L‐Kyn but markedly upregulated in L‐Kyn + CH‐treated neurons (Figure 5E). Overall, these results suggest that the neuroprotective effects of L‐Kyn are AhR‐dependent and that AhR could negatively modulate the NLRP3 protein expression.

### 3.6 Systemic Administration of L‐Kyn Attenuated Neuronal Pyroptosis in Rats

**FIGURE 6:**
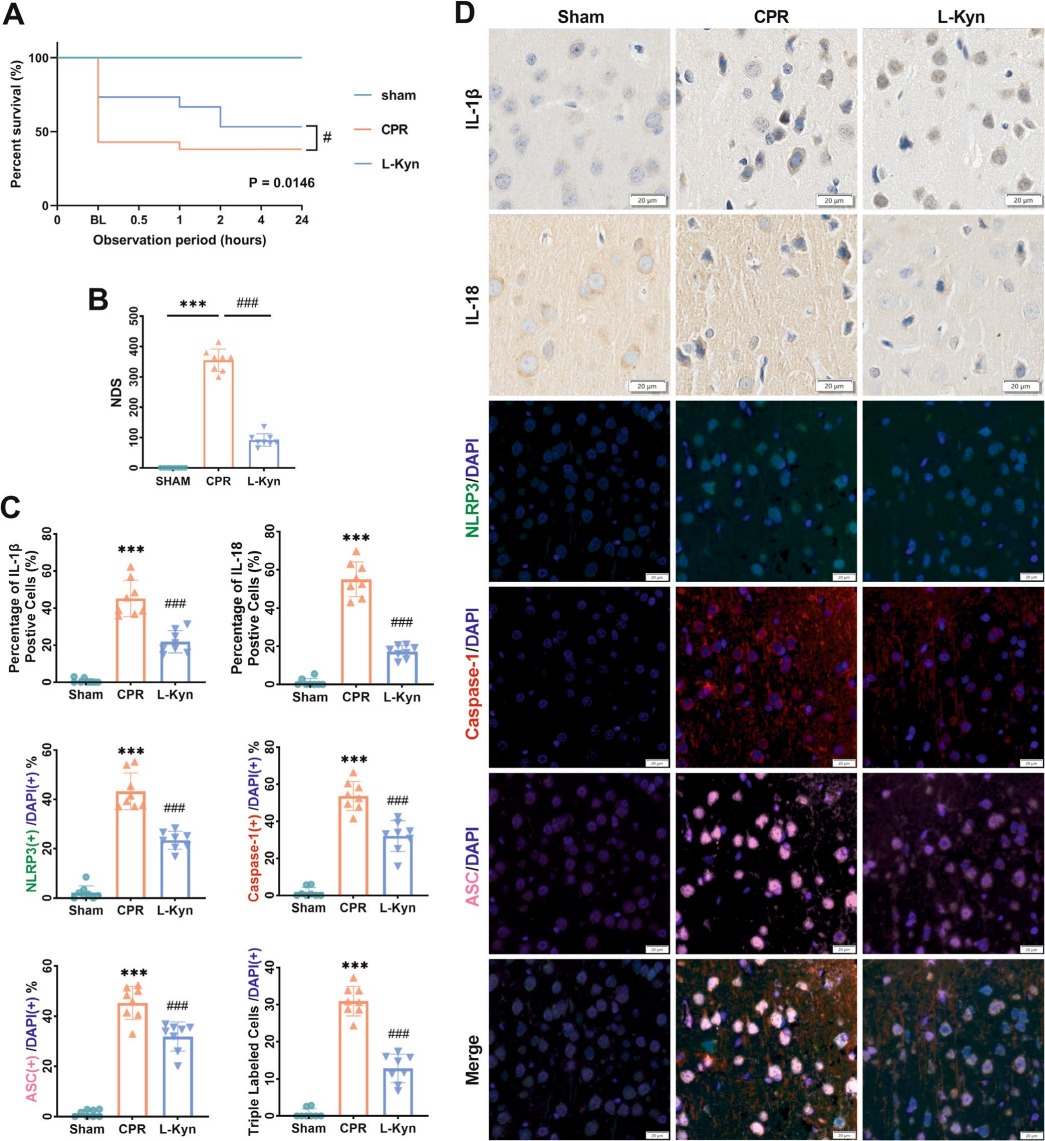
L‐Kyn attenuated NLRP3‐induced neuronal pyroptosis in rats. (A) Cumulative survival analysis (a Kaplan–Meier curve). (B) NDS of survived rats at 24 h post‐resuscitation (*n* = 8). (C) Quantification of IL‐1β, IL‐18, NLRP3, caspase‐1, ASC, and triple labeled cells in the cerebral cortex (*n* = 8). (D) Representative images of IL‐1β and IL‐18 immunohistochemistry staining and NLRP3, caspase‐1, and ASC immunofluorescence staining in the frontal cortex (scale bar = 20 μm). Data are expressed as mean ± SD. *p < 0.05, **p < 0.01, and ***p < 0.001 versus sham group; ^#^ *p* < 0.05, ^##^ *p* < 0.01, and ^###^ *p* < 0.001 versus CPR group.

Given the RNA‐sequencing analysis (Figure S6) and the findings in vitro, we conducted a second animal experiment focusing on pyroptosis‐related molecules. Forty‐three rats were assigned to CA/CPR (*n* = 35) or sham surgery (*n* = 8). Among CA/CPR animals, 14 received L‐Kyn injection and 21 received vehicle (Figure S2B). Continuous 24‐h survival monitoring showed that L‐Kyn significantly improved cumulative survival rates (log‐rank test, *p* = 0.0146; Figure 6A) and ROSC success (Table S4). For HR, the HR decreased in the L‐Kyn rats versus the CPR rats at 0.5‐ and 1‐h post‐resuscitation, while this change was restored in L‐Kyn rats at 2‐ and 4‐h post‐resuscitation (Table S4). For MAP, at 0.5‐, 1‐, 2‐, and 4‐h post‐resuscitation, the MAP was lower in CPR rats than in sham rats, whereas MAP in L‐Kyn rats was higher than in CPR rats (Table S4). Furthermore, the NDS increased significantly in both CPR and L‐Kyn rats compared to sham rats, yet decreased markedly in L‐Kyn rats versus CPR rats (Figure 6B).

To verify the NLRP3 inflammasome assembly and pro‐inflammatory intracellular contents release, we performed immunohistochemistry and immunofluorescence staining on the cerebral cortices of rats at 24 h post‐resuscitation (Figure 6C,D). The co‐localization of NLRP3, caspase‐1, and ASC indicated intracellular inflammasome activation. Quantitative analysis showed that the percentages of NLPR3 (+), caspase‐1 (+), ASC (+), triple labeled, interleukin (IL)‐1β (+), and IL‐18 (+) cells increased remarkably in CPR rats versus sham rats, which were all decreased in L‐Kyn‐treated rats versus CPR rats. Furthermore, the cerebral cortical mRNA levels of NLRP3, ASC, caspase‐1, GSDMD, IL‐1β, and IL‐18 were all significantly upregulated in CPR rats versus sham rats but significantly downregulated in L‐Kyn‐treated rats versus CPR rats (Figure S7). Collectively, these observations suggest that systemic administration of L‐Kyn partially inhibits CA/CPR‐induced neural pyroptosis and improves the neurological outcomes.

## 4 Discussion

Over the past decade, the fields of microbiology and neurology have become more closely entwined due to advances in sequencing and metabolomics techniques, giving rise to the concept of the microbiota‐gut‐brain axis [6, 7, 29]. Previous evidence indicates that the gut microbiota plays an indispensable role in neuropsychiatric and neurological diseases, including depression, schizophrenia, epilepsy, stroke, and brain injury [6, 7, 30]. However, thus far, the biological role of gut microbiota in PCABI has not yet been studied. Here, we pretreated rats with Abx to investigate the effects of gut microbiota dysbiosis on PCABI. Intriguingly, we found that Abx unexpectedly improved the survival and neurological outcomes in Abx/CPR rats. Although Abx may not be a practical long‐term therapeutic approach for PCABI, our findings provide novel insights into the role of gut microbiota in the pathophysiology of PCABI.

### 4.1 Gut Microbial Composition Impacts Intestinal Permeability

The effects of the gut microbiota in the microbiota‐gut‐brain axis are usually mediated by microbial metabolites [10], particularly the three most‐studied categories of short‐chain fatty acids, secondary bile acids, and Trp metabolites [12]. Gut microbial metabolites should cross at least two parts to reach the brain, namely the intestinal epithelial barriers and the BBB. Alterations in gut microbial composition significantly impact microbial metabolite synthesis. Our study revealed that Abx remarkably reduced the total bacterial load and microbial biodiversity. However, it did not achieve complete eradication of bacteria, possibly due to the emergence of antibiotic‐resistant bacteria. Consequently, Abx triggered a reconfiguration of the gut microbial composition.

Typically, *Firmicutes* and *Bacteroides* are the predominant phyla of bacteria [31]. However, in Abx/CPR rats, the two most predominant phyla of bacteria are *Firmicutes* and *Proteobacteria*. At the genus level, Abx decreased the relative abundances of the genera *Escherichia‐Shigella, Akkermansia, Ruminococcus_1*, *Bacteroides*, and *Parabacteroides*, and increased the relative abundances of the genera *Bifidobacterium* and *Pseudomonas*. Notably, the *Akkermansia genus*, the sole known member of the *Verrucomicrobia phylum* in human feces, is an abundant member of the human gut microbiota [32]. *Akkermansia c*olonizes the mucus layer on the surface of the intestinal mucosa, sustaining the IEB integrity [33]. *Ruminococcus_1, Escherichia‐Shigella, Bacteroides*, and *Parabacteroides* are tryptophanase‐encoding bacteria, producing IP metabolites like skatole, indolelactic acid, IAA, tryptamine, IA, IPA, and IAAld [34, 35], which are crucial for maintaining IEB integrity [35].

Therefore, Abx‐induced alterations in the microbial composition would damage the IEB integrity. Furthermore, PICRUSt2 analysis of the gut microbiota and KEGG enrichment analysis of fecal metabolites indicate a significant upregulation of Trp metabolism post‐resuscitation. Overall, these findings strongly suggest that Abx enhances the gut permeability to metabolites and that microbial Trp metabolism is intricately associated with the pathophysiology of PCABI.

### 4.2 Possible Mechanisms of KP Activation After Early Resuscitation

Trp, an essential amino acid, must be obtained through the diet and has a vital role in immune regulation [11]. The KP is the major route of Trp metabolism in the mammalian brain [7], and KP dysregulation is strongly associated with neurological disorders [14]. Notably, a recent study by Loretz et al. reported that KP dysregulation occurs in PCABI [17], but the underlying mechanism would be elucidated by this study.

Consequently, we integrated multi‐omics analysis and in vitro mechanistic experiments to elucidate the underlying mechanisms driving KP activation and its therapeutic modulation in PCABI. The results showed that the microbial composition was altered post‐resuscitation, increasing the neuroprotective metabolite Kyn in the feces, circulation, and brain successively. CA/CPR also induced cerebral KAT2 upregulation, increasing the production of downstream neuroprotective metabolites (KynA, XA). Furthermore, the gene expression of AhR, the receptor targeted by these neuroprotective metabolites, was also upregulated post‐resuscitation. It is reasonable to propose that the elevated peripheral Kyn levels post‐resuscitation are part of a complex self‐protective response to severe PCABI.

However, Abx shifted the phenotype of microbial Trp metabolism from the IP toward KP, further elevating the levels of Kyn in feces, circulation, and the brain. Moreover, Abx further increased cerebral AhR gene expression. Huai et al. [25] reported that AhR could negatively regulate NLRP3‐inflammasome activation. Immunohistochemistry, immunofluorescence, western blotting, and qRT–PCR results consistently showed that L‐Kyn alleviated NLRP3‐induced pyroptosis, and our in vitro experiments reveal that the neuroprotective action of L‐Kyn is AhR‐dependent.

Taken together, these findings demonstrate that Abx improves the neurological outcomes in PCABI may largely be due to the microbial Trp metabolite‐mediated AhR activation, which in turn inhibits NLRP3‐induced pyroptosis.

### 4.3 Potential Therapeutic Targets for PCABI in KP

We hypothesize that the KP‐related enzymes, specific microbiomes, and metabolites are candidate therapeutic targets for PCABI. Presently, enzyme‐based therapies are in development, and Ro‐61‐8048 (the most extensively used KMO inhibitor) has shown neuroprotective efficacy in focal or global ischemia. Its precursor, JM6, is neuroprotective in Huntington's disease and Alzheimer's disease with a reduction in cerebral 3‐hydroxykynurenine (neurotoxic) and QA [14]. While our study indeed found that CA/CPR upregulated the hippocampal KMO gene expression levels in rats, whether KMO inhibitors have a similar neuroprotective effect in PCABI warrants further validation.

Moreover, the specific bacterial species currently reported to metabolize Trp mostly convert Trp into IP metabolites, although the specific bacterial species involved in the KP are poorly defined. Recent studies have reported that *Pseudomonas* is converting Trp to Kyn [36] and 3‐hydroxykynurenine [37]. In this study, 62 genera had significant differential abundance, of which 17 genera displayed correlations with biochemical markers of cerebral injury (Figure S8A). A correlation analysis of these differentially abundant genera with fecal KP metabolites suggests that 21 genera might participate in KP (Figure S8B), which will be validated in our future studies.

Additionally, based on the results of this study, the AhR activation via L‐Kyn supplementation may also be a promising therapeutic strategy.

### 4.4 Limitation

This study has several limitations meriting consideration. Firstly, the rats were sacrificed at 24 h post‐resuscitation, and most of the testing parameters were assessed at a solitary time point. Hence, to comprehensively assess long‐term outcomes and temporal dynamics of these indexes, extended observation periods coupled with multiple experimental measurement points are necessary. Secondly, the metabolites in feces, serum, and plasma were detected by an untargeted method; a targeted approach allows absolute quantitative analysis of metabolites. Lastly, the AhR‐target regulations were solely conducted in vitro, while the effects of the L‐Kyn/AhR/NLRP3 pathway still require further validation in vivo.

## 5 Conclusions

In conclusion, we believe that the microbiota‐gut‐brain axis is critically involved in the pathogenesis of PCABI. Our results demonstrate that L‐Kyn may attenuate PCABI by inhibiting NLRP3‐induced pyroptosis via AhR activation, and that AhR activation via L‐Kyn supplementation may be a potential therapeutic strategy for PCABI. These findings provide additional insights into novel diagnostic and therapeutic strategies for PCABI.

## Author Contributions

W.H. and M.Z. designed and supervised the study; C.W., M.D., S.Y., S.X., and S.W. conducted the experiments; C.W., Z.Z., Y.C., and Y.Z. analyzed the data; C.W. drew the graphs and wrote the manuscript. All authors have read and approved the final manuscript.

## Ethics Statement

All animal procedures in the present study were approved by the Institutional Animal Care and Use Committee of Zhejiang Center of Laboratory Animals (ZJCLA‐IACUC‐20020032) and were conducted according to the United States Public Health Service's Policy on Humane Care and Use of Laboratory Animals.

## Conflicts of Interest

The authors declare no conflicts of interest.

## Supporting information


Data S1

## Data Availability

The sequencing clean data have been deposited in the National Center for Biotechnology Information database (PRJNA881761 and PRJNA881026). Further inquiries can be directed to the corresponding authors.
