# Supplementary material for: Gut Microbial Tryptophan Metabolism Is Involved in Post‐Cardiac Arrest Brain Injury via Pyroptosis Modulation
Source: CNS Neurosci Ther. 2025 Apr 22;31(4):e70381. doi: 10.1111/cns.70381 (PMC12012640; doi:10.1111/cns.70381)
Supplement: Supplementary file 1 — Data S1 [file CNS-31-e70381-s001.docx]

**Online supplement for:**

**Gut microbial tryptophan metabolism is involved in post-cardiac arrest brain injury via pyroptosis modulation.**

Chenghao Wu^2, 3#^, Mengyuan Diao^1#^, Shuhang Yu^4^, Shaosong Xi^1^, Zhipeng Zheng^6^, Yang Cao^5^, Shuai Wang^1^, Ying Zhu^1^, Mao Zhang^2^*, Wei Hu^1^*

^1^ Department of Critical Care Medicine, Affiliated Hangzhou First People’s Hospital, School of Medicine, Westlake University, Hangzhou, Zhejiang, China

^2^ Department of Emergency Medicine, Second Affiliated Hospital, Zhejiang University School of Medicine, Hangzhou, Zhejiang, China

^3^ Anesthesia Center of Critical Care Research, Department of Anesthesia, Critical Care and Pain Medicine, Massachusetts General Hospital, Harvard Medical School, Boston, MA, USA

^4^ Department of Intensive Care Unit, Second Affiliated Hospital, Zhejiang University School of Medicine, Hangzhou, Zhejiang, China

^5^ Department of Neurosurgery, Affiliated Hangzhou First People’s Hospital, School of Medicine, Westlake University, Hangzhou, Zhejiang, China

^6^ Department of Pulmonary and Critical Care Medicine, Sir Run Run Shaw Hospital, Zhejiang University School of Medicine, Hangzhou, Zhejiang, China.

^#^These authors contributed equally

Please send correspondence to:

*Wei Hu

Department of Critical Care Medicine, Affiliated Hangzhou First People’s Hospital, School of Medicine, Westlake University, No. 261 Huansha Road, Hangzhou 310006, China. Email: huwei@hospital.westlake.edu.cn

*Mao Zhang

Department of Emergency Medicine, Second Affiliated Hospital, Zhejiang University School of Medicine, No. 88 Jiefang Road, Hangzhou 310009, China. E-mail: z2jzk@zju.edu.cn

**Supplementary material:**

Supplementary methods

Figures S1–S8

Tables S1–S4

References

## Methods

**Animals grouping**

Part 1 (Supplementary Fig. S2A): Male adult Sprague–Dawley rats (400–500 g; Zhejiang Center of Laboratory Animals, China) were randomly distributed into sham, Abx, CPR, and Abx/CPR groups. The Abx and Abx/CPR groups were fed with ampicillin sodium salt (1 g/L; A9518, Sigma-Aldrich, USA), neomycin sulfate (1 g/L; N6386, Sigma-Aldrich), vancomycin hydrochloride (500 mg/L; VL0657, Eli Lilly, USA), and metronidazole (1 g/L; M3761, Sigma-Aldrich) in drinking water for four weeks to eradicate the gut microbiota ^1^. In contrast, the sham and CPR groups were fed with autoclaved sterile water. The CPR and Abx/CPR groups subsequently established a 7-min asphyxia CA model, while the sham and Abx groups only received surgical operation. Part 2 (Supplementary Fig. S2B): Male adult Sprague–Dawley rats (400–500 g; Zhejiang Center of Laboratory Animals) were randomized into sham, CPR, and L-Kyn groups. The sham group only received surgical operation, while the CPR and L-Kyn groups established the CA model. At the start of resuscitation, rats in CPR and L-Kyn groups were intravenously injected with saline and L-Kyn (50 mg/kg), respectively. Initial group sizes were determined based on pilot studies indicating CA/CPR procedural mortality rates (~40-60%). Larger cohorts were allocated to groups with higher expected mortality to ensure ≥8 survivors/group at 24 h for endpoint analyses. All mortality events, regardless of timing, were included in survival curves.

**Rat model of CA**

We generated the asphyxia-CA/CPR model as described previously ^2^, with minor modifications. In brief, asphyxia-CA was induced by stopping ventilation after intravenous infusion of vecuronium, typically leading to CA within 4 min, which was characterized by the absence of arterial pulse and a MAP of < 25 mmHg. After identifying CA, no treatment was administered for 7 min. Then, CPR was promptly initiated by intravenous infusion of adrenaline (20 μg/kg), ventilation with pure oxygen, and chest compressions (250 compressions/min). Additional doses of adrenaline were administered at 2-min intervals until ROSC, defined as a MAP > 60 mmHg persisted for a minimum of 10 min; typically, rats achieve ROSC within 2 min after CPR implementation. If the rats failed to achieve ROSC after 10 min, resuscitation was terminated. At 4-h post-resuscitation, all catheters were removed, surgical incisions were closed, and the rats were returned to separate cages with ad libitum food and water. (Supplementary Fig. S2C)

**Monitoring and sample collection**

Electrocardiogram, MAP, rectal temperature, and EtCO_2_ were continuously monitored throughout the procedure. Blood samples were drawn and centrifugated at baseline and 1, 4, and 24 h post-resuscitation, and after centrifugation, the sera were stored at -80°C. The animals were sacrificed at 24-h post-resuscitation, and the feces, frontal cortex, and hippocampus were harvested for subsequent experiments. (Supplementary Fig. S2C)

**NDS**

The NDS was evaluated at 24-h post-resuscitation, according to a validated scoring system ^3^. The NDS ranged from 0 (normal) to 500 (brain death) and was independently scored by two assessors who were blinded to the study.

**ELISA**

To quantify the serum concentrations of NSE and S100b of rats, ELISA kits (RX302363 & RX301878, RUIXIN, China) were used following the manufacturer’s instructions.

**Histological examination**

Freshly harvested hippocampus and frontal cortex were fixed in 10% neutral-buffered formalin overnight, stepwise dehydrated, and then embedded in paraffin. Paraffin-embedded tissue blocks were sliced into 5-μm thick sections for hematoxylin–eosin (H&E), immunohistochemistry, and immunofluorescence staining.

For H&E staining, briefly, the slices were prepared by dewaxing, staining with H&E, dehydrating, and mounting with neutral resin. In each microscopic field (400× magnification), the number of viable neurons was determined under an optical microscope (BX3, Olympus, Japan). The criteria for viable neurons include a visible nucleus and an intact cytoplasm. Neurons with eosinophilic cytoplasm, cytoplasmic vacuolation, perikaryal shrinkage, and nuclear pyknosis were excluded ^4^.

For immunohistochemistry staining, the sections were subjected to dewaxing and antigen retrieval. After immersing in 3% H_2_O_2_ to suppress endogenous peroxidase activity, the slices were permeabilized with 0.025% Triton X-100 and blocked with 10% goat serum. Subsequently, the slices were incubated with anti-IL-18 (1:1000; ab191860, Abcam, UK) or anti-IL-1β (1:2500; ab9722, Abcam) primary antibodies at 4°C overnight, before incubating with HRP-conjugated secondary antibodies (1:200; GB1213, Servicebio, China). The peroxidase reaction was developed with diaminobenzidine, before counterstaining the sections with hematoxylin. Images were obtained using an optical microscope (Olympus).

For triple-labeling immunofluorescence staining, the slices were dewaxed, subjected to antigen retrieval, permeabilized with 0.2% Triton X-100 and then incubated with Blocking Buffer (ab126587, Abcam). Subsequently, the sections were incubated at 4°C overnight with anti-NLRP3 primary antibodies (1:500; PA5-79740, Invitrogen, USA), before incubating with FITC-conjugated secondary antibody (1:100; GB22303, Servicebio) at room temperature for 1 h. Then, after performing antigen retrieval for the second time, the slides were incubated with anti-caspase-1 primary antibodies (1:1000; PA5-87526, Invitrogen) at 4°C overnight, before incubating with CY5-conjugated secondary antibody (1:800; GB27303, Servicebio) at room temperature for 1 h. In the same way, after performing antigen retrieval for the third time, the slides were incubated with anti-ASC primary antibodies (1:1000; ab180799, Abcam) at 4°C overnight, before incubating with CY3-conjugated secondary antibody (1:400; GB21303, Servicebio) at room temperature for 1 h. The nuclei were counterstained with DAPI. Finally, the sections were photographed using a fluorescence microscope (IX83, Olympus), and the resulting images were merged and analyzed by ImageJ (NIH, USA).

**RNA-sequencing and qRT****–PCR**

The total RNA was extracted from frozen tissues using an RNAeasy™ Animal RNA Isolation Kit (Beyotime, China) following the manufacturer’s protocol and quantified by NanoDrop (ThermoFisher). For the methodological details of RNA-sequencing, see Fatima et al. ^5^. Data processing was conducted using an online platform provided by LC-Bio (https://www.omicstudio.cn/analysis).

To measure the levels of mRNA expression, qRT–PCR was performed using the ChamQ Universal SYBR qPCR Master Mix (Vazyme, China) and ABI QuantStudio 6 Flex Real-Time PCR system following the manufacturer’s instructions. The primers (Tsingke, China) used in this study are listed in Supplementary Table S1. The results were represented as fold changes of the threshold cycle (Ct) value relative to controls using the 2 ^−ΔΔCt^ method following normalization to β-actin.

**Microbial community analyses**

Microbial DNA isolation and 16S rDNA sequencing were performed following the method of Peng et al. ^6^. Data processing was conducted using an online platform provided by LC-Bio (https://www.omicstudio.cn/analysis).

**Metabolomic analyses**

For the methodological details of the untargeted metabolomic analysis, see Zhang et al. ^7^ The XCMS software (ThermoFisher) was used for data processing. The Human Metabolome Database (HMDB) and KEGG were used to identify metabolites. For the methodological details of the targeted metabolomic analysis, see Ren et al. ^8^. The collected raw data were processed with Xcalibur software (ThermoFisher). Standard curves were constructed from least-squares linear regression analysis using the peak area ratio of the derivatized individual standard versus the nominal calibrator concentration (Supplementary Table S2). The quantification of samples was calculated identically.

**Cell culture experiments**

The primary neuronal culture and OGD/R were established with a minor modification of the model used in the study by Sun et al. ^9^. Briefly, primary neuronal cultures were prepared from the brains of fetal rats on embryonic day 17. The isolated hippocampi were digested with 0.25% trypsin/EDTA (T1320, Solarbio, China) for 12 min at 37°C. To inactivate the trypsin, tissue pieces were physically dissociated and neurons were suspended in Dulbecco’s Modified Eagle’s Medium (DMEM; 11995500, Gibco, USA) containing 20% fetal bovine serum (10099141C, Gibco). The neurons were centrifuged and re-suspended in the standard neuron culture medium: Neurobasal Medium (2110349, Gibco) with 2% B-27 (17504044, Gibco), 0.5 mM glutamine (35050061, Gibco), 1% penicillin and 1% streptomycin (SV30010, HyClone, USA). Then, the cell suspension was seeded on 6-well and 96-well plates pre-coated with Poly-D-lysine (50 μg/mL, ST508, Beyotime). Half the volume of the medium was replaced twice weekly. After 10-day incubation, the cultures were subjected to 4-h OGD by changing the medium to glucose-free DMEM (MA0585, Meilunbio, China) and transferring the cultures into a hypoxia chamber (95% N_2_/5% CO_2_). At the end of OGD, the cells were returned to the standard neuron culture medium, and treated with different doses (0, 1, 10, 100, and 1000 μM) of L-Kyn (S5839, Selleck, USA) or (0, 1, 10, 100, and 1000 nM) CH (C8124, Sigma-Aldrich), and incubated in a normoxic chamber for 24 h. Cells incubated in the standard neuron culture medium under normoxic conditions were used as controls. Following the instructions, cell viability was evaluated using a Cell Counting Kit-8 (CK-04, Dojindo, Japan), and cellular apoptosis was detected using an Annexin V-APC/PI apoptosis kit (AT107, MULTI, China).

**Western blotting**

The total protein of primary cultured neurons was lysed using RIPA lysis buffer (FD009, Fdbio, China). The protein concentration of each sample was determined using a BCA Protein Quantitative Kit (DQ111-01, TransGen, China), and equal amounts of protein (20 μg/well) were loaded onto gels for SDS-PAGE. The proteins were electrophoresed until they had sufficiently separated and were then transferred to PVDF membranes (0.22 μm; Millipore, USA). The membranes were blocked with 5% skim milk at room temperature for 1 h and then incubated with anti-NLRP3 antibody (1:1000; ab263899, Abcam) and anti-β-actin antibody (1:1000; 4970S, CST, USA) at 4°C overnight. Following incubation, the membranes were processed with anti-rabbit HRP-linked antibody (1:3000; 7074S, CST) at room temperature for 1 h. The BeyoECL Star chemiluminescence reagent kit (P0018AS, Beyotime) was used to observe the exposed membranes, and the blot densities were quantified with ImageJ.

## Figures

**Fig. S1 Overview of the Trp metabolic pathway**

**
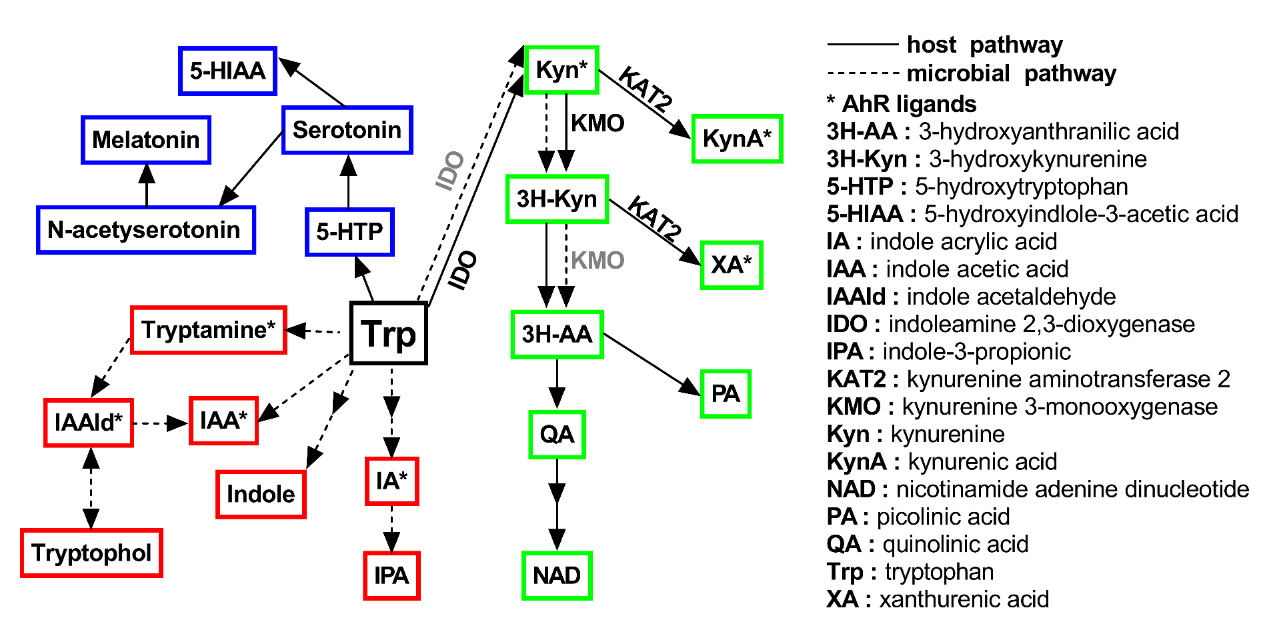
**

Trp metabolism follows three major pathways: serotonin pathway (blue), indole pathway (red), and kyn pathway (green).

**Fig. S2 Flow diagram and experimental protocol**


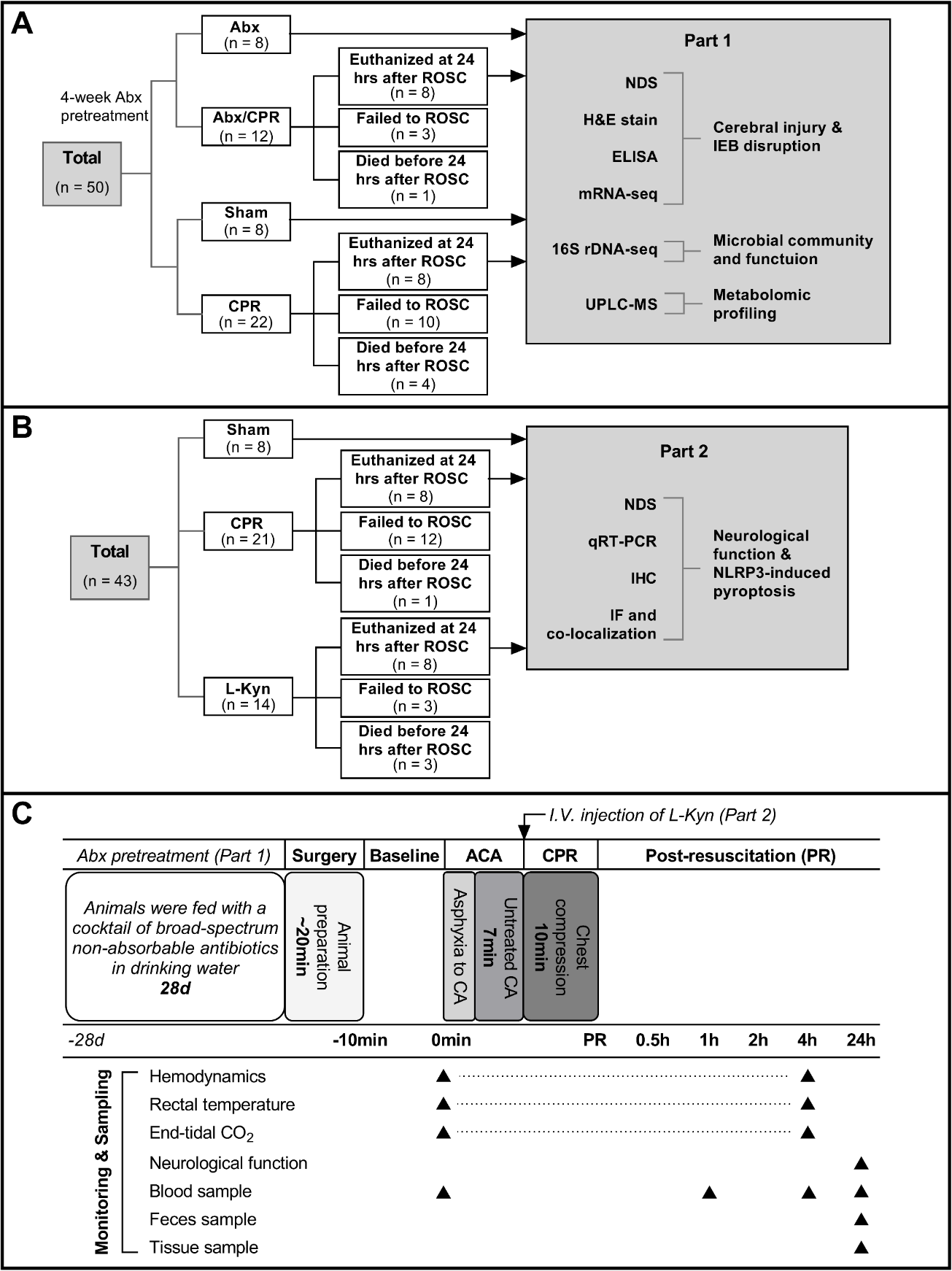


Experimental procedure, measurements and samplings during baseline, ACA, CPR, and PR. To ensure sufficient survivors (n=8/group) for endpoint analyses at 24 h, we allocated larger initial cohorts to groups with higher anticipated procedural mortality (e.g., untreated CPR controls). All animals undergoing CA/CPR (regardless of survival time) were included in the Kaplan-Meier analysis. *UPLC-MS* ultraperformance liquid chromatography-mass spectrometer, *ACA* asphyxial cardiac arrest

**Fig. S3 Microbial diversity was altered in Abx/CPR rats. (Data supporting Fig 2)**

**
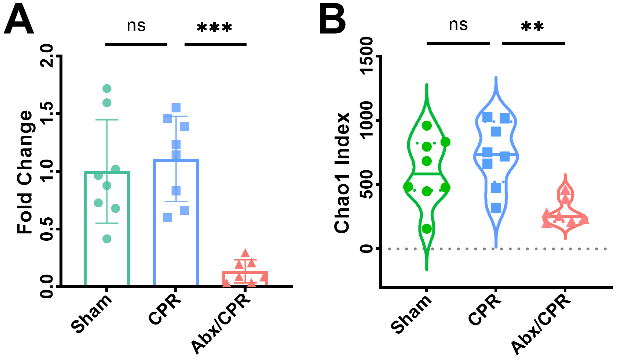
**

**(A)** Total bacterial load (n = 7–8). **(B)** Microbial α-diversity (Chao1 index; n = 7–8). Data are expressed as mean ± SD or median with IQR. **P < 0.01 and ***P < 0.001 vs indicated group; ns not significant

**Fig. S4 Functional difference analysis of gut microbiota. (Data supporting Fig 2)**

**
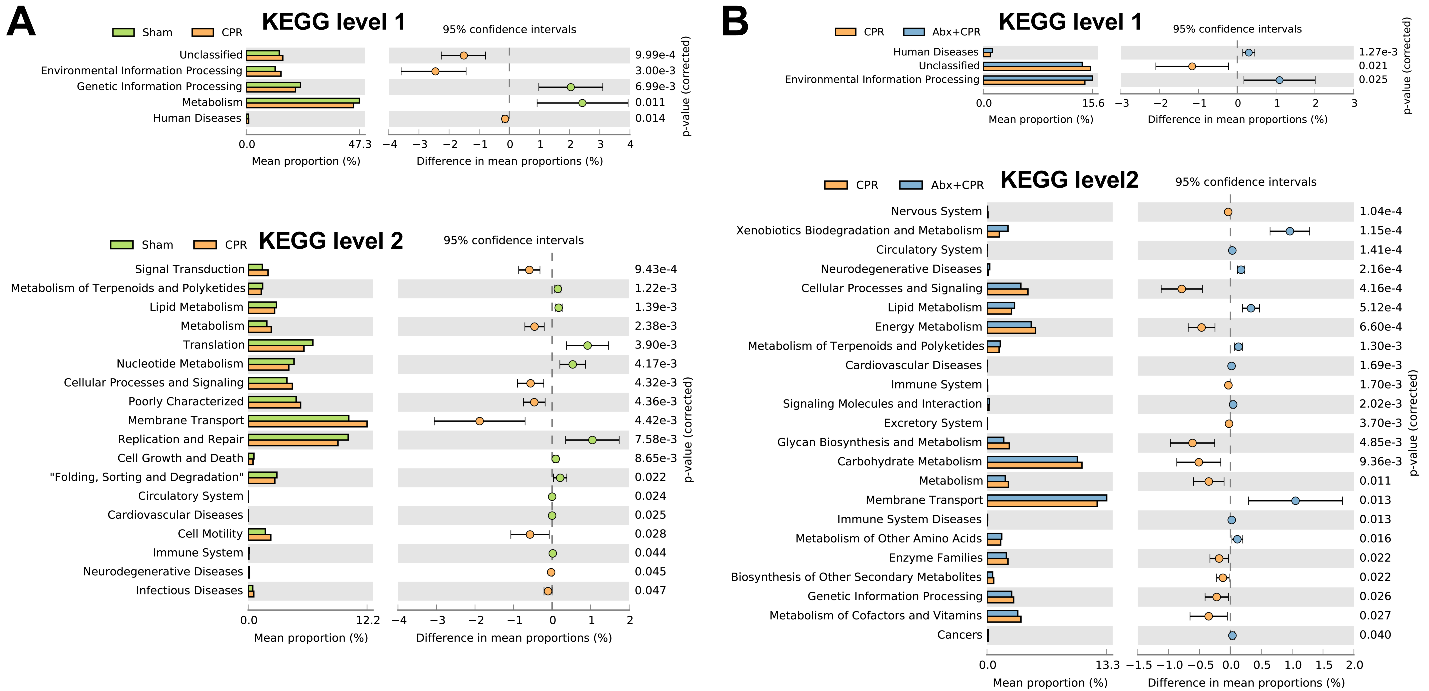
**

**(A)** Predicted functional content at KEGG level 1 and level 2 between sham and CPR groups. **(B)** Predicted functional content at KEGG level 1 and level 2 between CPR and Abx/CPR groups. Data are expressed as mean proportion and its 95% confidence intervals only with the difference of mean proportions > 0.12%, a ratio of proportion > 2, and *P* < 0.01 between two groups.

**Fig. S5 Hippocampal Trp metabolic profiling (Data supporting Fig 4)**

**
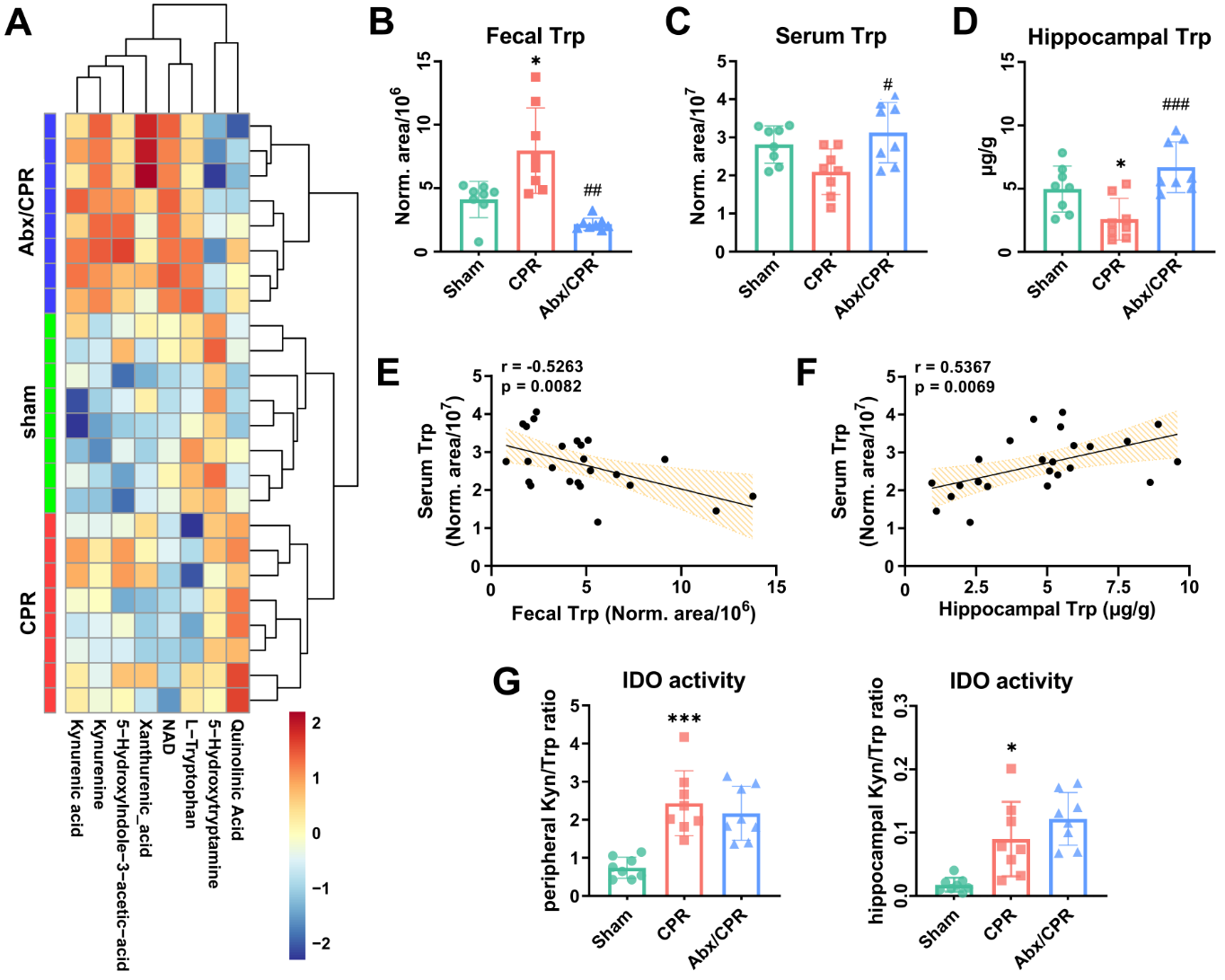
**

**(A)** Hippocampal Trp metabolic profiling (n = 8). **(B, C, D)** Alterations in fecal Kyn, serum Kyn and hippocampal Kyn (n = 8) **(E)** Pearson’s correlation analysis between fecal Trp and serum Trp (n = 24). **(F)** Pearson’s correlation analysis between hippocampal Trp and serum Trp (n = 24) **(G)** The peripheral and hippocampal IDO activity (n = 8). Data are presented as mean ± SD. **P* < 0.05, ***P* < 0.01, and ****P* < 0.001 vs sham group; ^#^*P* < 0.05, and ^###^*P* < 0.001 vs CPR group.

**Fig. S6** **The rat hippocampus exhibited significant neuroinflammation post-resuscitation.**

**
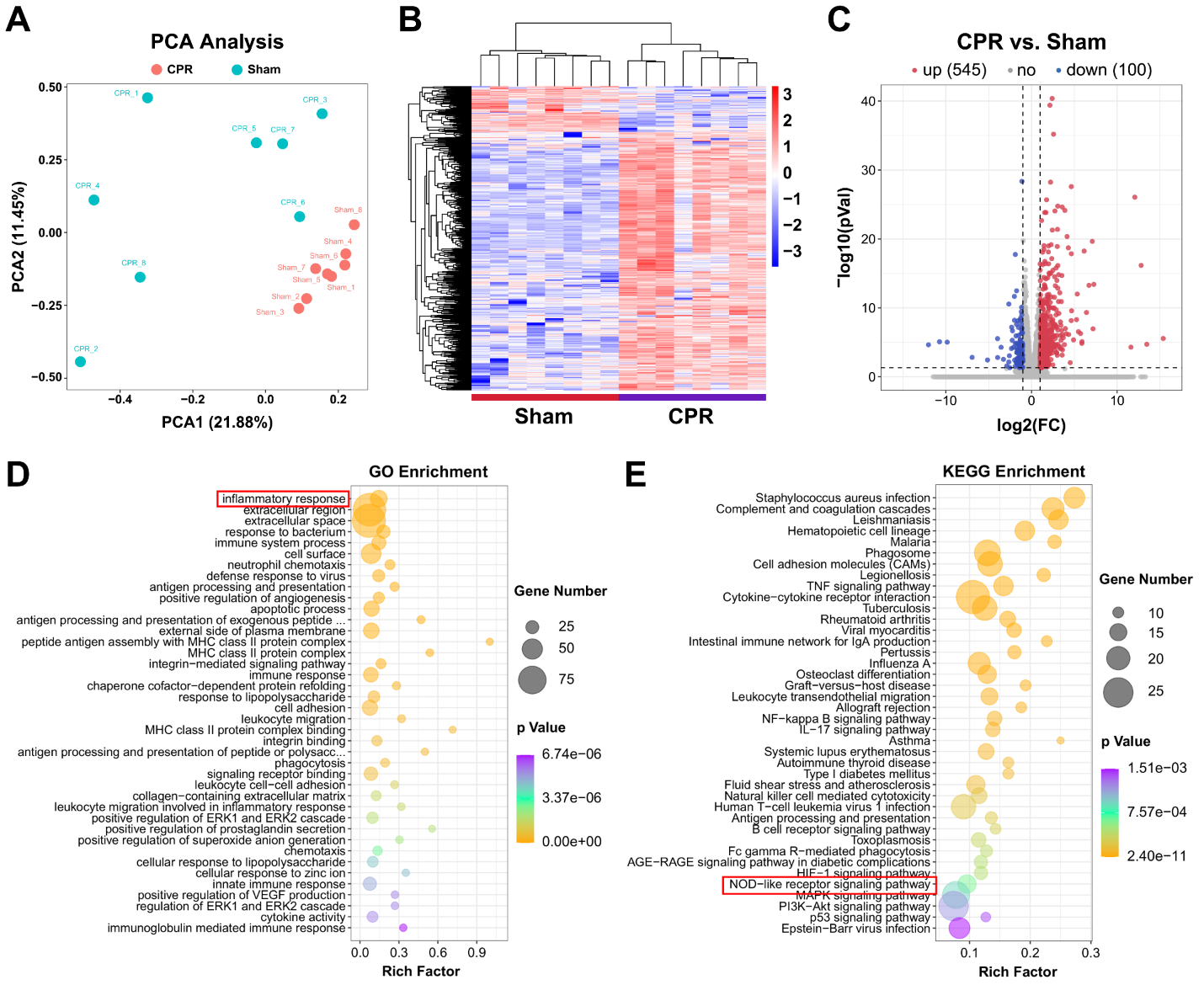
**

**(A)** A principal component analysis plot of the differentially expressed genes in the CPR and sham rats were constructed from transcriptome analysis (n = 8). **(B)** Heatmap of differentially expressed genes (fold change > 2 or < 0.5, p < 0.05; n = 8). **(C)** Volcano plot of change in gene expression (n = 8). **(D, E)** Statistics of GO and KEGG enrichment (n = 8).

**Fig. S7 The expression of pyroptosis-related genes in the cortex**

**
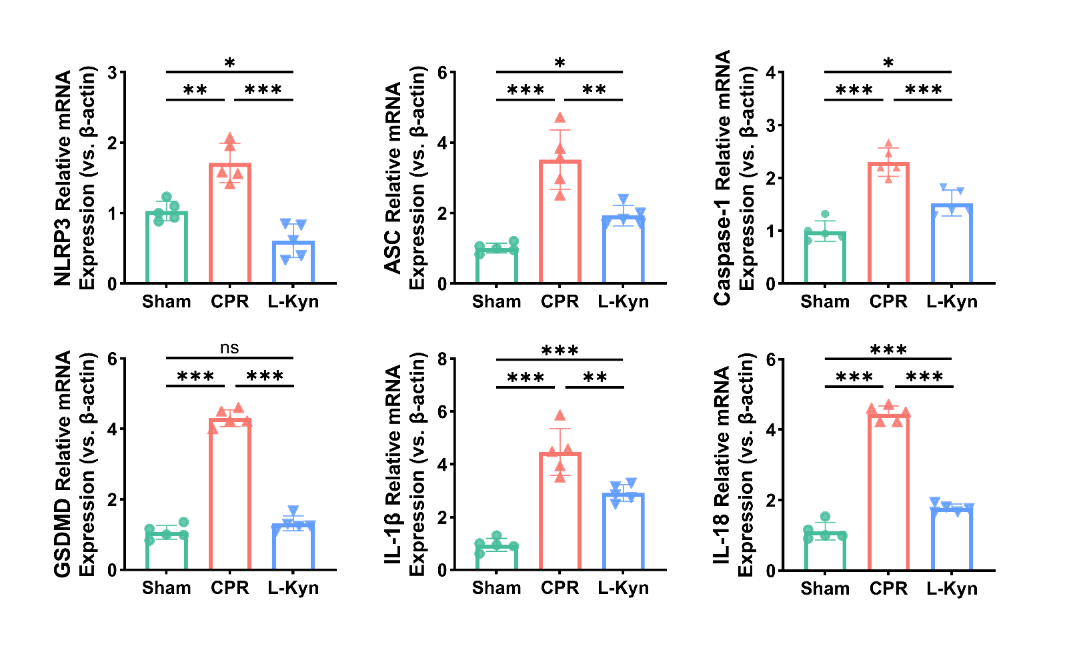
**

Relative mRNA expression of NLRP3, ASC, caspase-1, GSDMD, IL-1β, and IL-18 (n = 5). Data are presented as mean ± SD. **P* < 0.05, ***P* < 0.01, and ****P* < 0.001.

**Fig. S8 Potential therapeutic targets for PCABI (specific microbiomes)**

**
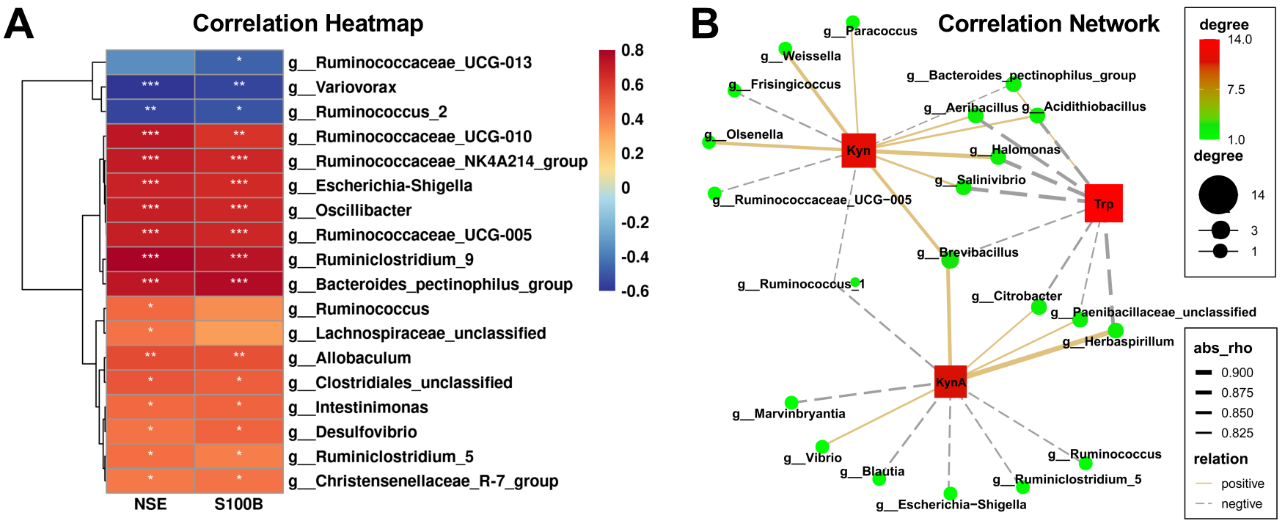
**

**(A)** Correlation analysis between biochemical markers and gut microbiota (Spearman’s ρ > 0.5 or < -0.5 and *P* < 0.05; n = 8). **(B)** Correlation analysis between Kyn pathway metabolites and gut microbiota (Spearman’s ρ > 0.8 or < –0.8 and *P* < 0.01; n = 8).

## Tables

**Table S1 Primer sequences used for qRT–PCR**

| Gene | Primer | Sequence | Size |
| --- | --- | --- | --- |
| Rat β-Actin | Forward | 5′-AGATCAAGATCATTGCTCCTCCT-3′ | 174 bp |
|  | Reverse | 5′-ACGCAGCTCAGTAACAGTCC-3′ |  |
| Rat NLRP3 | Forward | 5′-ACAACTCACCCAAGGAGGAAG-3′ | 150 bp |
|  | Reverse | 5′-TTGAGAAGAGACCTCGGCAG-3′ |  |
| Rat ASC | Forward | 5′-GCAGATGGACCCCATAGACC-3′ | 107 bp |
|  | Reverse | 5′-GCTCCTGTATGCCCATGTCT-3′ |  |
| Rat Caspase-1 | Forward | 5′-GGAGCTTCAGTCAGGTCCATC-3′ | 113 bp |
|  | Reverse | 5′-ATGCGCCACCTTCTTTGTTC-3′ |  |
| Rat GSDMD | Forward | 5′-CCACCAAAGCCGGAAGAAGA-3′ | 212 bp |
|  | Reverse | 5′-CCCCGATGGAATGGAGTACG-3′ |  |
| Rat IL-1β | Forward | 5′-CAGCTATGGCAACTGTCCCT-3′ | 138 bp |
|  | Reverse | 5′-CATCTGGACAGCCCAAGTCA-3′ |  |
| Rat IL-18 | Forward | 5′-CAAAAGAAACCCGCCTGTGT-3′ | 199 bp |
|  | Reverse | 5′-AGGTGGATTCATTTCCTCAAAGG-3′ |  |
| Rat KAT2 | Forward | 5′-CATCTGGCTGTCAAGACGGT-3′ | 121 bp |
|  | Reverse | 5′-CAGCCCAGTGGTTTCATTGC-3′ |  |
| Rat KMO | Forward | 5′-GCTCATGCCATCGTCCCATT-3′ | 114 bp |
|  | Reverse | 5′-AGGAAGGCACACACTAAGATCA-3′ |  |
| Rat AhR | Forward | 5′-TGCGTCTTCCACTATCCAAGATT-3′ | 100 bp |
|  | Reverse | 5′-GAACTCAGCTCGGTCTTCTGTAT-3′ |  |
| Rat ARNT | Forward | 5′-ATCCCAACATCAGTGCAGATCAG-3′ | 202 bp |
|  | Reverse | 5′-TGACGAGAAATCTGGGCCAATAT-3′ |  |

**Table S2 Standard curves used for metabolite quantification**

| metabolite | regression equation | linear range (μg/L) | correlation coefficient r |
| --- | --- | --- | --- |
| L-Tryptophan | Y=7.01×10^3^X-8.92×10^4^ | 250–1500 | 0.9998 |
| L-Kynurenine | Y=8.20×10^2^X-1.32×10^4^ | 25–500 | 0.9954 |
| Kynurenic acid | Y=1.12×10^4^X-6.55×10^3^ | 5–100 | 0.9991 |
| Xanthurenic acid | Y=9.37×10^3^X-7.35×10^4^ | 50–800 | 0.9998 |
| Quinolinic acid | Y=1.80×10^2^X-1.63×10^4^ | 500–2000 | 0.9978 |
| Serotonin | Y=3.55×10^3^X+8.15×10^2^ | 0.5–10 | 0.9952 |
| 5-HIAA | Y=2.99×10^3^X+7.24×10^2^ | 10–250 | 0.9998 |
| NAD^+^ | Y=1.30×10^2^X-3.32×10^3^ | 100–1000 | 0.9973 |

**Table S3. Parameters comparison among the four groups (Part 1)**

| Parameters | Time points | Sham | Abx | CPR | Abx/CPR |
| --- | --- | --- | --- | --- | --- |
| Body weight, g | Baseline | 432.3 ± 21.3 | 433.4 ± 22.8 | 438.3 ± 18.6 | 421.0 ± 17.6 |
| Time form asphyxia to CA, s | Baseline | — | — | 185.6 ±17.0 | 198.1 ± 16.2^c^ |
| Time required for ROSC, s | Baseline | — | — | 86.4 ± 21.52 | 43.78 ± 11.3**^c^** |
| ROSC rate, % | Baseline | — | — | 54.55 | 75.00 |
| Heart rate, beats/min | Baseline | 322.9 ± 20.4 | 323.4 ± 16.8 | 330.1 ± 15.7 | 326.9 ± 15.8 |
|  | PR 0.5 h | 331.5 ± 10.8 | 328.0 ± 7.1 | 304.3 ± 35.4^b^ | 331.4 ± 9.3^c^ |
|  | PR 1 h | 322.3 ± 13.1 | 312.8 ± 9.5 | 310.3 ± 32.0 | 322.1 ± 15.3 |
|  | PR 2 h | 321.0 ± 14.6 | 317.9 ± 11.7 | 331.0 ± 22.4 | 321.0 ± 16.1 |
|  | PR 4 h | 318.0 ± 10.5 | 323.4 ± 11.3 | 334.4 ± 12.9 | 330.6 ± 14.3 |
| MAP, mmHg | Baseline | 122.0 ± 6.8 | 115.8 ± 7.5 | 126.8 ± 4.8 | 121.9 ± 3.7 |
|  | PR 0.5 h | 129.4 ± 4.1 | 119.4 ± 5.2 | 72.6 ± 13.9^b^ | 69.2 ± 11.3 |
|  | PR 1 h | 126.0 ± 3.2 | 115.1 ± 5.1 | 77.3 ± 11.9^b^ | 90.4 ± 10.8^c^ |
|  | PR 2 h | 120.8 ± 4.1 | 115.4 ± 5.7 | 89.6 ± 3.7^b^ | 97.5 ± 11.9 |
|  | PR 4 h | 122.3 ± 6.0 | 115.3 ± 5.2 | 85.0 ± 10.2^b^ | 98.8 ± 10.5^c^ |
| End-tidal CO_2_, mmHg | Baseline | 34.25 ± 2.32 | 34.0 ± 2.51 | 36.05 ± 1.40 | 34.25 ± 2.56 |
|  | PR 0.5 h | 32.75 ± 2.12 | 34.13 ± 2.23 | 52.11 ± 11.98^b^ | 21.89 ± 7.41^c^ |
|  | PR 1 h | 32.63 ± 2.50 | 33.00 ± 2.56 | 53.00 ± 10.45^b^ | 25.00 ± 4.12^c^ |
|  | PR 2 h | 32.0 ± 2.56 | 35.88 ± 3.48 | 55.00 ± 7.75^b^ | 28.38 ± 2.88^c^ |
|  | PR 4 h | 33.50 ± 4.00 | 33.50 ± 2.67 | 37.25 ± 4.17 | 28.75 ± 2.66^c^ |

Physiological variables are measured at baseline and at 0.5, 1, 2, and 4 h post-resuscitation or sham operation. Data are expressed as mean ± SD.  ^a^*P* < 0.05 sham *vs* Abx, ^b^*P* < 0.05 sham *vs* CPR, ^c^*P* < 0.05 CPR *vs* Abx/CPR.

**Table S4. Parameters comparison among the three groups (Part 2)**

| Parameters | Time points | Sham | CPR | L-Kyn |
| --- | --- | --- | --- | --- |
| Body weight, g | Baseline | 437.6 ± 16.7 | 436.7 ± 27.4 | 434.4 ± 26.9 |
| Time form asphyxia to CA, s | Baseline | — | 205.5 ±16.9 | 214.8 ± 26.2 |
| Time required for ROSC, s | Baseline | — | 107.4 ± 27.0 | 87.0 ± 26.7 |
| ROSC rate, % | Baseline | — | 42.86 | 71.43^b^ |
| Heart rate, beats/min | Baseline | 326.1 ± 11.5 | 328.2 ± 12.9 | 332.8 ± 10.5 |
|  | PR 0.5 h | 323.3 ± 14.1 | 323.4 ± 11.0 | 251.0 ± 35.1^b^ |
|  | PR 1 h | 328.3 ± 12.6 | 329.5 ± 13.7 | 286.9 ± 44.7^b^ |
|  | PR 2 h | 326.6 ± 20.1 | 328.0 ± 15.3 | 314.2 ± 23.3 |
|  | PR 4 h | 327.9 ± 19.0 | 328.4 ± 15.1 | 322.6 ± 17.7 |
| MAP, mmHg | Baseline | 123.6 ± 6.2 | 121.9 ± 7.6 | 127.5 ± 6.3 |
|  | PR 0.5 h | 125.0 ± 8.7 | 68.6 ± 3.5^a^ | 86.3 ± 11.1 ^b^ |
|  | PR 1 h | 123.5 ± 10.4 | 71.3 ± 3.1^a^ | 93.5 ± 13.8^b^ |
|  | PR 2 h | 120.3 ± 3.2 | 75.4 ± 3.5^a^ | 100.6 ± 8.6^b^ |
|  | PR 4 h | 121.1 ± 8.5 | 82.4 ± 5.4^a^ | 97.8 ± 6.8^b^ |

Physiological variables are measured at baseline and at 0.5, 1, 2, and 4 h post-resuscitation or sham operation. Data are expressed as mean ± SD. ^a^*P* < 0.05 sham *vs* CPR, ^b^*P* < 0.05 CPR *vs* L-Kyn.

## References

1. Wu Q, Xu Z, Song S, Zhang H, Zhang W, Liu L*, et al.* Gut microbiota modulates stress-induced hypertension through the HPA axis. *Brain Res Bull* 2020, **162:** 49-58.

2. Huang K, Wang Z, Gu Y, Hu Y, Ji Z, Wang S*, et al.* Glibenclamide Is Comparable to Target Temperature Management in Improving Survival and Neurological Outcome After Asphyxial Cardiac Arrest in Rats. *Journal of the American Heart Association* 2016, **5**(7).

3. Hendrickx HH, Rao GR, Safar P, Gisvold SE. Asphyxia, cardiac arrest and resuscitation in rats. I. Short term recovery. *Resuscitation* 1984, **12**(2)**:** 97-116.

4. Helfaer MA, Ichord RN, Martin LJ, Hurn PD, Castro A, Traystman RJ. Treatment with the competitive NMDA antagonist GPI 3000 does not improve outcome after cardiac arrest in dogs. *Stroke* 1998, **29**(4)**:** 824-829.

5. Fatima I, Chen G, Botchkareva NV, Sharov AA, Thornton D, Wilkinson HN*, et al.* Skin Aging in Long-Lived Naked Mole-Rats is Accompanied by Increased Expression of Longevity-Associated and Tumor Suppressor Genes. *J Invest Dermatol* 2022.

6. Peng LY, Shi HT, Tan YR, Shen SY, Yi PF, Shen HQ*, et al.* Baicalin inhibits APEC-induced lung injury by regulating gut microbiota and SCFA production. *Food Funct* 2021, **12**(24)**:** 12621-12633.

7. Zhang S, Sun F, Zhang C, Zhang M, Wang W, Zhang C*, et al.* Anthocyanin Biosynthesis and a Regulatory Network of Different-Colored Wheat Grains Revealed by Multiomics Analysis. *J Agric Food Chem* 2022, **70**(3)**:** 887-900.

8. Ren Q, Cheng L, Guo F, Tao S, Zhang C, Ma L*, et al.* Fisetin Improves Hyperuricemia-Induced Chronic Kidney Disease via Regulating Gut Microbiota-Mediated Tryptophan Metabolism and Aryl Hydrocarbon Receptor Activation. *J Agric Food Chem* 2021, **69**(37)**:** 10932-10942.

9. Sun B, Ou H, Ren F, Huan Y, Zhong T, Gao M*, et al.* Propofol inhibited autophagy through Ca(2+)/CaMKKβ/AMPK/mTOR pathway in OGD/R-induced neuron injury. *Molecular medicine (Cambridge, Mass)* 2018, **24**(1)**:** 58.
